# Management of Mixed Mechanism Glaucoma Secondary to NewColorIris Implant Using an Ab Externo Xen Gel Stent

**DOI:** 10.1155/crop/9742946

**Published:** 2026-05-08

**Authors:** Roshun Sangani, Param Shukla, Miriam M. Habiel

**Affiliations:** ^1^ Institute of Ophthalmology & Visual Science, Rutgers New Jersey Medical School, Newark, New Jersey, USA, rutgers.edu

**Keywords:** iris prosthesis, mixed mechanism glaucoma, NewColorIris implant, off-label, Xen Gel Stent

## Abstract

**Background and Aims:**

We present a case of mixed mechanism glaucoma following NewColorIris implant that was successfully treated using an off‐label use of Xen Gel Stent.

**Methods:**

A retrospective review of the patient′s medical records was conducted following the acquisition of informed consent.

**Results:**

A 42‐year‐old female with a history of bilateral cosmetic iris implants presented to the emergency room due to corneal decompensation and a secondary mixed mechanism glaucoma caused by implant iris chafing in her left eye. The patient underwent prosthetic iris implant removal and had persistently elevated intraocular pressures (IOPs) postoperatively. She underwent a subsequent off‐label Xen Gel Stent implantation which has successfully controlled her eye pressures without the need for supplementary antiglaucoma medications for 13 months. This off‐label use of the Xen Gel Stent helped the patient maintain excellent IOP and satisfied the patient′s cosmetic preferences.

**Conclusion:**

Xen Gel Stent may be a safe and effective option to treat secondary glaucoma due to cosmetic iris implants.

## 1. Introduction

Iris implants were initially developed to treat conditions such as congenital or traumatic aniridia, oculocutaneous albinism, and iris colobomas [1]. Several implants are currently available on the global market, including the CustomFlex ArtificialIris (HumanOptics AG) and the BrightOcular implant (Stellar Devices). NewColorIris implants (Kahn Medical Devices, Panama City, Panama) were popularly implanted as well; however, several reports indicate that the company is no longer in business. Of the aforementioned implant types, the CustomFlex ArtificialIris is the only FDA‐approved implant to date for this purpose. Recently, these iris implants have been marketed for cosmetic purposes, such as the popular NewColorIris implants [2]. Unfortunately, these cosmetic iris implants have known complications including corneal decompensation, uveitis, cystoid macular edema, and uncontrolled intraocular pressure (IOP) [3]. In a case series of 14 eyes from Hoguet et al., seven had elevated IOP. Of those seven eyes, three eyes consequently needed trabeculectomy, and three eyes needed glaucoma drainage implant placement [4]. The complications from these implants require a multidisciplinary approach involving treatment of uveitis, glaucoma, cataract formation, and corneal decompensation. The pathogenesis of the elevated IOP in these patients is thought to be multifactorial due to the implant′s direct contact with angle structures within the anterior chamber and iris pigment liberation within the anterior segment [5]. The Xen Gel Stent is approved to manage elevated IOP for patients with open‐angle glaucoma, pigmentary glaucoma, or pseudoexfoliative glaucoma who are not controlled on maximum tolerated medical therapy [6]. In this report, we present a unique case where our patient underwent iris implant removal followed by off‐label Xen Gel Stent insertion to successfully control mixed mechanism glaucoma refractory to maximal tolerated medical therapy.

## 2. Methods

All the patients allowed personal data processing, and informed consent was obtained from all individual participants included in the study. This report does not contain any personal information that could lead to the identification of the patient. A retrospective chart review was performed to gather pertinent information.

## 3. Case Report

A 42‐year‐old woman with no significant past medical history sought treatment at the emergency department (ED) at University Hospital in Newark, New Jersey, due to acute‐on‐chronic blurry vision and left eye pain for the past few months. The patient′s ocular history was significant for bilateral artificial iris implants 5 years prior to presentation in the ED. She had consulted an outside ophthalmologist who started the patient on COMBIGAN and latanoprost eye drops for elevated IOP in her left eye 2 weeks prior to this ED visit.

At the time of presentation, visual acuity was 20/20 in the right eye and 20/400 with pinhole correction to 20/150 in the left eye. IOP was 14 mmHg in her right eye and 18 mmHg in her left. No relative afferent pupillary defect was observed. External examination was normal in both eyes. Slit lamp examination was notable for a centered artificial iris implant in both eyes and diffuse 2+ corneal edema with bullae and endothelial pigment in the left eye. Video S1 shows the NewColorIris implant in the patient′s left eye while she is blinking. The patient consequently received a Descemet membrane endothelial keratoplasty (DMEK) along with surgical peripheral iridotomy. The procedure was completed without any complications, and her glaucoma medications were continued. However, the patient continued to have persistently elevated IOP postoperatively and was referred to the glaucoma clinic for management.

**Figure 1:**
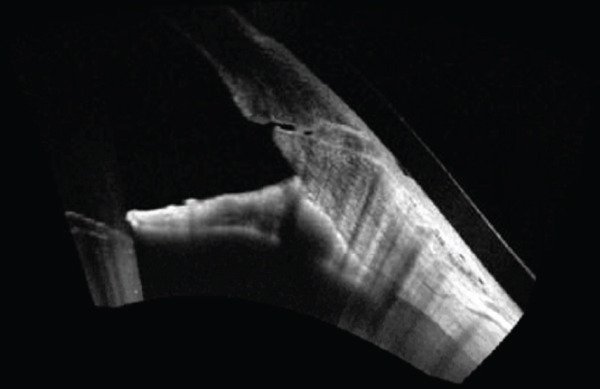
Peripheral anterior synechiae. Anterior segment OCT of the patient′s left eye displaying peripheral anterior synechiae with high iris insertion.

**Figure 2:**
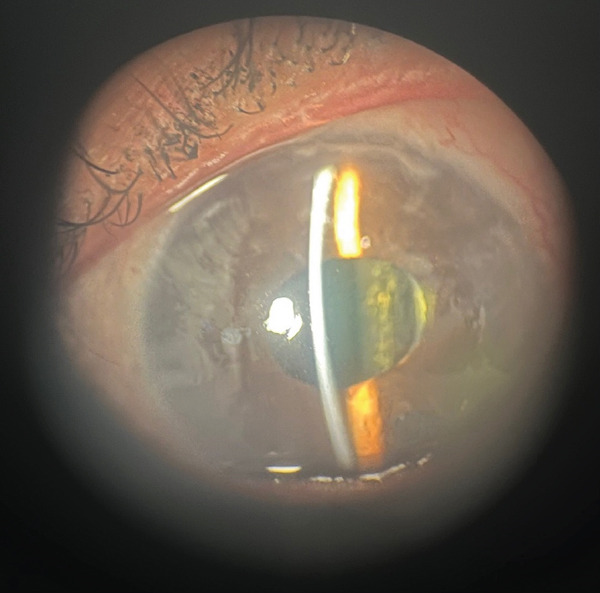
Iris atrophy. This is an external photo of the patient′s iris after the implant was removed using viscodissection, and the peripheral anterior synechiae were released. Multiple areas of iris atrophy are present superiorly and nasally, signified by the opaque color.

At presentation to our glaucoma clinic, the patient was postop Month 2.5 from her DMEK and had an elevated IOP of 34 mmHg in her left eye. Her angle was open to the scleral spur in the right eye and closed in the left eye on gonioscopy. A mild posterior subcapsular cataract was noted in the left eye. Cup‐to‐disk ratio was noted to be 0.6 OD and 0.5 OS. The patient wanted to keep her cosmetic implants and declined removal. Maximal tolerated topical glaucoma medications were started, and adequate IOP control was achieved for 6 months. At the 6‐month follow‐up visit, the patient had an elevated IOP of 29 mmHg in her left eye, and the patient agreed to proceed with implant removal. The patient underwent iris prosthesis removal along with goniosynechialysis in her left eye. During the procedure, the iris implant was found to have caused posterior and peripheral anterior synechiae (Figure 1). The implant was removed using viscodissection, and the peripheral anterior synechiae were released using micrograsper forceps. Multiple areas of iris atrophy were also noted on examination (Figure 2). The patient tolerated the procedure well but still had persistently elevated IOP postoperatively. As a result, Diamox was added to her medical regimen in addition to maximally tolerated topical glaucoma medications, and this was enough to normalize the IOP pressures. Due to persistent corneal edema, the patient underwent an additional DMEK approximately 4 months after iris prosthesis removal. The patient tolerated the procedure well, but still maintained persistently elevated IOP after repeat DMEK. Options for glaucoma filtration surgery were discussed with the patient including an off‐label use of Xen Gel Stent. The patient was very concerned about her cosmetic appearance after glaucoma surgery and did not want a large implant. The patient elected to undergo Xen Gel Stent implantation for IOP control.

Approximately 7 months after iris prosthesis removal and 3 months after the patient′s second DMEK, the patient underwent Xen Gel Stent (45 *μ*m diameter lumen) implantation with Mitomycin C. The procedure was started with a subtenon injection of Mitomycin C followed by a small conjunctival peritomy. Next, the gel stent was implanted via an ab externo approach. Care was made to ensure that the implant had 1 mm in the anterior chamber, 2 mm in the sclera, and 3 mm in the superior subconjunctival quadrant. Intraoperative gonioscopy confirmed the Xen entry site at the level of the trabecular meshwork in the anterior chamber. At the conclusion of the case, the stent was functioning well, and a bleb was observed to be forming. At the postop Day 1 visit, the IOP was 12 mmHg, and all IOP‐lowering drops along with Diamox were discontinued. At the postop Week 1, postop Week 2, postop Month 1, and postop Month 3 visits, the IOP ranged between 11 and 17 mmHg. Five months after insertion of the stent, a decision to pursue cataract surgery in the left eye with the placement of an intraocular lens was made due to the presence of a visually significant cataract. At the time of her most recent visit, postop Month 13 from Xen Gel Stent implantation with Mitomycin C and 20 months after initial iris prosthesis removal, the patient had an IOP of 16 mmHg in her left eye along with pinhole visual acuity to 20/30 off glaucoma drops.

## 4. Discussion

**Figure 3:**
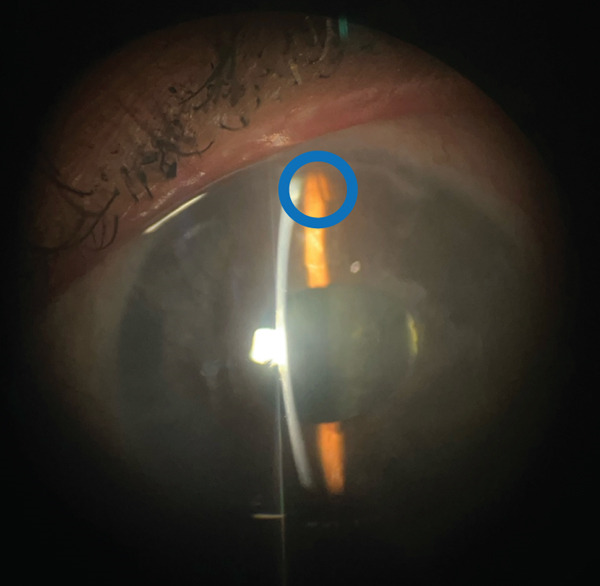
Stent shadow. An external photo using the slit lamp which highlights a shadow of the Xen Gel Stent. The stent is the thin shadow in the center of the blue circle.

**Figure 4:**
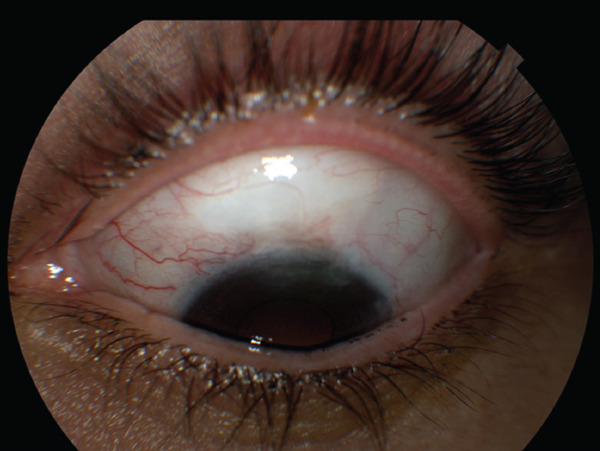
Successful implantation. This is an external photo of the bleb that forms superiorly when the Xen Gel Implant is successfully placed and draining.

Artificial iris implants have been utilized for over 2 decades to treat symptoms associated with iris defects secondary to aniridia, trauma, or iatrogenic complications [7]. An irregular iris and pupil can result in increased glare, reduced contrast sensitivity, and depth of focus alterations [7]. The CustomFlex ArtificialIris is the only FDA‐approved iris implant to treat congenital aniridia and other iris defects in the United States [8]. Postoperatively, patients experienced less glare, improved contrast sensitivity, and satisfactory cosmesis [7, 9]. Unfortunately, some companies such as NewColorIris implant (Kahn Medical Devices, Panama City, Panama) and BrightOcular implant (Stellar Devices) have marketed their devices to cosmetically change iris colors in otherwise healthy eyes. These procedures have been associated with glaucoma, synechiae, corneal decompensation, cataract formation, and retinal detachments [3, 7]. Mayer et al. illustrated that up to 25.5% of eyes with artificial iris implantations can show the aforementioned complications [3]. Another literature review of 18 eyes with the NewColorIris implant from Sikder et al. found that in patients who suffer complications from implantation, up to 77.7% of them present with complications such as hyphema, elevated IOP, and blurred vision within the first postoperative month [5]. The increased rate of adverse complications from iris implants, particularly glaucoma, has been documented previously. Galvis et al. reported in a literature review of 128 eyes with cosmetic iris implants that 88 (68.8%) of the eyes required explantation secondary to complications. The most common complication was secondary glaucoma which was observed in 59 eyes (46.1%), followed by severe endothelial cell loss with or without corneal decompensation which was present in 44 eyes (34.4%) [10]. Galvis and his team believe the complications are due to the design of the device and its implantation in the anterior chamber. They postulate that the etiology may be related to compression of the trabecular meshwork causing chronic inflammation, contact between the implant′s flaps, and contact with the iris leading to atrophy and pigment dispersion [10]. In our patient, her other eye revealed significant implant instability and movement with each blink, further supporting the likelihood of increased implant iris chafing, creating inflammation and uveitis. The movement of the implant in the right eye is clearly visualized in Video S1. Ghaffari et al. published a retrospective case series in which they observed 19 of 24 eyes (79.2%) requiring removal of their NewColorIris or BrightOcular artificial iris [11]. The most common indication for removal was elevated IOP (64.7%) and corneal edema (47.1%) [11]. Alarmingly, there were signs of moderate‐to‐advanced glaucomatous optic nerve damage in 9 of the 24 eyes (37.5%) [11]. Considering that artificial iris implantation is a cosmetic procedure, the presence of serious complications with poor visual outcomes is concerning. Previous reports have discussed the use of trabeculectomies or tube shunts to control secondary glaucoma in these patients. In our patient with mixed mechanism glaucoma secondary to cosmetic iris implantation, we were able to successfully control her IOP with an off‐label use of the Xen Gel Stent. The patient′s glaucoma was attributed to two distinct pathophysiologic mechanisms, consistent with the diagnosis of mixed mechanism glaucoma. The first mechanism involved angle closure, identified on gonioscopic examination during initial presentation. The second mechanism was ocular hypertension and secondary glaucoma resulting from mechanical irritation of the iris by the cosmetic iris prosthesis. This chronic chafing led to the formation of anterior synechiae and chronic inflammation, subsequently obstructing trabecular meshwork outflow and contributing to elevated IOP. After conducting a literature review on April 16, 2024, utilizing PubMed, Google Scholar, and Web of Science using the keywords “Xen Gel Stent,” “iris prosthesis,” and “iris implant,” we did not find any prior reports of Xen Gel Stent implantation after iris prosthesis–induced ocular hypertension. Of note, the Xen Gel Stent is not approved for cases of closed‐angle glaucoma which our patient may have initially had. However, after iris implant removal and subsequent goniosynechialysis, the angle was open, and the Xen Gel Stent was able to be safely placed in a space away from Descemet′s membrane and the iris. A shadow of the implanted stent can be visualized in the superior aspect of the eye (Figure 3) along with the bleb formed from Xen implantation (Figure 4). After discussing multiple surgical options with the patient, the patient opted for a minimally invasive glaucoma surgery that would control her pressures while providing an adequate cosmetic result. The patient strongly desired the least conspicuous implant to ensure a good cosmetic outcome. Additional benefits included a quick recovery process and fewer postop visits than traditional glaucoma surgeries. While not the most important, another request from the patient was for an uncomplicated medicine routine. The patient′s discomfort with complex topical therapy warranted a procedure which provided a similar chance at IOP reduction when comparing Xen Gel Stent to valved shunt procedures [6]. For these reasons, shared decision‐making led to a consensus that the Xen Gel Implant may give the patient the best shot at achieving her treatment goals. At the 13‐month mark after Xen insertion, her IOPs have been stable between 11 and 19 mmHg without the aid of any glaucoma drops. The patient was able to maintain good IOP control with a preferred cosmetic outcome and without the need for any pressure‐lowering drops for more than a year after the procedure.

We believe our patient′s increased IOP was initially due to a component of secondary angle closure with consequent trabecular meshwork dysfunction with an additional inflammatory component. Our case report suggests that the Xen Gel Stent may be an effective option for combatting cosmetic iris implant–induced elevated IOP after iris prosthesis removal and could be considered an alternative to traditional incisional glaucoma surgery such as drainage implant placement or trabeculectomy.

## Funding

No funding was received for this manuscript.

## Disclosure

All authors attest that they meet the current ICMJE criteria for authorship. The order of authorship has been confirmed by all authors. All authors have read and approved the final version of the manuscript. Param Shukla had full access to all of the data in this study and takes complete responsibility for the integrity of the data and the accuracy of the data analysis. Param Shukla affirms that this manuscript is an honest, accurate, and transparent account of the study being reported, that no important aspects of the study have been omitted, and that any discrepancies from the study as planned (and, if relevant, registered) have been explained.

## Conflicts of Interest

The authors declare no conflicts of interest.

## Supporting information


**Supporting Information** Additional supporting information can be found online in the Supporting Information section. Supporting Information. Video S1: Iris implant. This is a video of the patient′s left eye blinking before the iris implant was removed from her eye.

## Data Availability

The authors confirm that the data supporting the findings of this study are available within the article.
